# Evaluating the Influence of a G-Quadruplex Prone Sequence on the Transactivation Potential by Wild-Type and/or Mutant P53 Family Proteins through a Yeast-Based Functional Assay

**DOI:** 10.3390/genes12020277

**Published:** 2021-02-15

**Authors:** Paola Monti, Vaclav Brazda, Natália Bohálová, Otília Porubiaková, Paola Menichini, Andrea Speciale, Renata Bocciardi, Alberto Inga, Gilberto Fronza

**Affiliations:** 1 Mutagenesis and Cancer Prevention Unit, IRCCS Ospedale Policlinico San Martino, 16132 Genoa, Italy; paola.monti@hsanmartino.it (P.M.); paola.menichini@hsanmartino.it (P.M.); andrea.speciale@hsanmartino.it (A.S.); 2 Institute of Biophysics of the Czech Academy of Sciences, Královopolská 135, 61265 Brno, Czech Republic; vaclav@ibp.cz (V.B.); natalia.bohalova@ibp.cz (N.B.); o.porubiakova@gmail.com (O.P.); 3 Department of Food Chemistry and Biotechnology, Faculty of Chemistry, Brno University of Technology, Purkyňova 118, 61200 Brno, Czech Republic; 4 Department of Experimental Biology, Faculty of Science, Masaryk University, Kamenice 5, 62500 Brno, Czech Republic; 5 Department of Neurosciences, Rehabilitation, Ophthalmology, Genetics, Maternal and Child Health (DiNOGMI), University of Genoa, Largo P. Daneo 3, 16132 Genoa, Italy; bocciardi@unige.it; 6 Medical Genetics Unit, IRCCS Istituto Giannina Gaslini, via G. Gaslini 5, 16147 Genoa, Italy; 7 Laboratory of Transcriptional Networks, Department of Cellular, Computational and Integrative Biology, CIBIO, University of Trento, via Sommarive 9, 38123 Trento, Italy

**Keywords:** P53 family, yeast, G-quadruplex (G4) prone sequence, wild-type and mutant P53/P63 proteins, transactivation potential

## Abstract

P53, P63, and P73 proteins belong to the P53 family of transcription factors, sharing a common gene organization that, from the P1 and P2 promoters, produces two groups of mRNAs encoding proteins with different N-terminal regions; moreover, alternative splicing events at C-terminus further contribute to the generation of multiple isoforms. P53 family proteins can influence a plethora of cellular pathways mainly through the direct binding to specific DNA sequences known as response elements (REs), and the transactivation of the corresponding target genes. However, the transcriptional activation by P53 family members can be regulated at multiple levels, including the DNA topology at responsive promoters. Here, by using a yeast-based functional assay, we evaluated the influence that a G-quadruplex (G4) prone sequence adjacent to the p53 RE derived from the apoptotic *PUMA* target gene can exert on the transactivation potential of full-length and N-terminal truncated P53 family α isoforms (wild-type and mutant). Our results show that the presence of a G4 prone sequence upstream or downstream of the P53 RE leads to significant changes in the relative activity of P53 family proteins, emphasizing the potential role of structural DNA features as modifiers of P53 family functions at target promoter sites.

## 1. Introduction

The P53 family of transcription factors (TFs) is composed of P53, P63, and P73 proteins [1,2,3] that share an N-terminal transactivation domain, a central sequence-specific DNA-binding domain, and a C-terminal tetramerization domain. At the C-terminus, a sterile α motif domain (SAM), probably involved in protein–protein interactions, is present only in P63 and P73 proteins. These TFs, by inducing a plethora of target genes, can influence different cellular pathways including proliferation, apoptosis, DNA repair, angiogenesis, senescence, metabolism, and differentiation [4,5]. 

To further complicate the scenario, multiple P53, P63, and P73 isoforms are generated from mechanisms of alternative usage of promoters, splicing sites, and/or translation initiation sites [6]. All P53 family members can be transcribed starting from different promoters resulting in variants with different N-terminal sequences (i.e., full-length P53, ΔN40P53, TAP63, and TAP73 from the P1 promoter, and ΔN133P53, ΔN160P53, ΔNP63, and ΔNP73 from the P2 promoter). Moreover, C-terminal variations occur as a result of alternative splicing, giving rise to at least three different isoforms (i.e., α, β, γ for P53 and P63; α, β, γ, δ, ε, ζ, η for P73). 

Despite these similarities, the overlap in cellular functions between P53, P63, and P73 proteins is limited. *TP53* is the most frequently mutated gene in human sporadic cancers and *TP53* germ-line mutations are associated with the development of the cancer-prone Li-Fraumeni and Li-Fraumeni-like syndromes [7]. Conversely, the *TP63* gene, being critical for the correct development of ectodermal-derived tissues, is associated with the occurrence of a subset of ectodermal dysplasia syndromes (i.e., P63-associated disorders) due to *TP63* germ-line mutations [3,8,9]. Lastly, P73 contributes to neural and immune system functions but no genetic disorder has been linked to the gene [10,11], possibly because any such *TP73* mutation might produce severe defects that are embryonically lethal.

To regulate genes expression, the P53 family members, acting mainly as TFs, share a DNA response element (RE) which consists of two degenerate decameric sequences separated by a variable spacer [RRRCWWGYYY-(n)-RRRCWWGYYY (*R* = purine; *W* = A/T; *Y* = pyrimidine; *n* = 0–13)] [12,13,14]. Even though the DNA-binding specificities for P53, P63, and P73 appear comparable, differences for certain DNA sequences have been reported [15,16,17]. Moreover, gene expression and genome-wide occupancy studies reveal a partial overlap between gene networks of P53 family members [18]; in fact, many genes appeared to be exclusively targeted by one of the P53 family members [19,20,21,22]. 

Recently, it was shown that DNA topology can contribute to regulating P53 DNA affinity and specificity [23,24]. It was demonstrated that P53 protein binds to various DNA structures stabilized by DNA topological stress such as G-quadruplex (G4)-forming sequences, cruciforms, and other local DNA structures [24,25,26], as recently reviewed [27]. Furthermore, some P53 mutants have a high predisposition for binding to Transcription Start Site-associated G/C-rich regions, particularly G4s, which are prone to form non-B DNA structures [28].

The yeast *Saccharomyces cerevisiae* is a recognized model system for understanding different aspects of human biology. Thanks to the evolutionary conservation of basic components of the transcription machinery, many TFs, including P53 family proteins, when ectopically expressed in yeast cells can modulate the expression rates of reporter genes, acting through promoters engineered to contain cognate target REs [29,30,31]. Moreover, a particularly versatile approach based on the *Delitto Perfetto* technique is available in yeast to target the selected genomic loci through homologous recombination [32], leading to the development of a matrix of results where the functional interaction of TFs binding sites with other nearby cis-elements can also be evaluated [33].

Based on the assumption that DNA topology can be an important factor in establishing P53-dependent transactivation at target genomic sites, we previously evaluated the influence of a G4 DNA prone sequence in the proximity of a P53 RE on the transactivation potential of full-length and N-terminal truncated P53 α isoforms [34]. The results showed that a G4 prone sequence alone is not sufficient for transcriptional activation by the different P53 α isoforms (i.e., full length, ΔN40, ΔN133, and ΔN160); however, its presence in proximity to a P53 RE leads to significantly different fold changes in transcriptional activity and dynamics between the co-expressed P53 isoforms. 

Here, by using the same experimental approach in yeast, we extended this study to ΔN and TA α variants of P63 and P73 proteins. Moreover, since human germ-line *TP53* and *TP63* mutations are responsible for the development of specific genetic disorders and considering that *TP53* somatic mutations are frequent in human cancers, we also evaluated the effect of the presence of the same G4 prone sequence on the functional features of mutant P53 and P63 proteins. The results obtained highlight how the presence of a G4 prone sequence adjacent to a P53 RE impact the transcriptional activity of wild-type and mutant proteins from all P53 family members. 

## 2. Materials and Methods 

### 2.1. Yeast Strains and Media

A panel of isogenic reporter strains, which differ only in the presence and position of a P53 RE [from *PUMA* (p53 up-regulated modulator of apoptosis) target gene: 5′-CTGCAAGTCCTGACTTGTCC-3′] and a G4 prone sequence [from KSHV (Kaposi sarcoma-associated herpes virus): 5′-GGGGCGGGGGACGGGGGAGGGG-3′] both located upstream of the luciferase reporter gene (LUC1) were used [34,35,36]. The *PUMA* RE was selected in virtue of its moderate strength and for the presence of physiological G4 sequences around the RE in the human promoter. This panel includes yLFM-PUMA (PUMA RE), yLFM-KSHV (G4), yLFM-KSHV-PUMA (G4 upstream of PUMA RE), and yLFM-PUMA-KSHV (G4 downstream of PUMA RE) strains. Yeast cells were grown in YPDA medium (1% yeast extract, 2% peptone, 2% dextrose, 200 mg/L adenine) or selective medium (with or without 2% agar), containing dextrose or raffinose as a carbon source plus adenine (200 mg/L), but in the absence of tryptophan and/or leucine (Sigma-Aldrich, Saint Louis, MO, USA; Biokar Diagnostics, Allonne, France). Galactose (Sigma-Aldrich, Saint Louis, MO, USA) was added to the medium for the modulation of P53 family proteins expression under the inducible GAL1, 10 promoter [37]. Yeast manipulations were performed, as previously described [33,38].

### 2.2. Yeast Vectors

Wild-type P53 [full length (i.e., P53α corresponding to the well-known 393 amino-acids long protein) and ΔN40α], P63 (ΔNα and TAα) and P73 (ΔNα and TAα) proteins were expressed by yeast pTSG-based vectors (inducible GAL1,10 promoter, TRP1) [39,40,41] as well as mutant full-length P53 (i.e., R175H and R282W) and ΔNP63α (i.e., G134V and R204W) proteins [41,42]. For co-expression experiments vectors expressing wild-type full-length P53 and ΔNP63α under the constitutive ADH1 promoter [pLS76 (LEU2) and pLS-ΔNP63α (LEU2), respectively] were used along with the yeast plasmids described above [41,43]. Empty vectors pRS314 (TRP1) and pRS315 (LEU2) were available.

### 2.3. Yeast Functional Assay

Quantitative functional assays were performed according to the miniaturized protocol we developed [33,38]. Briefly, yeast transformants were grown at 30 °C in a selective medium containing 1% Galactose in order to modulate P53 family proteins expression; after 8 h of growth OD (600 nm) was measured. Twenty μL of cell suspension was transferred into a white plate and mixed with an equal volume of PLB buffer 2X (Promega Italia, Milan, Italy) to obtain the lysis of yeast cells. After 15 min of shaking at room temperature, firefly luciferase substrate (20 μL, Bright Glo, Promega) was added. Luciferase activity was measured using a multilabel plate reader (Mithras LB940, Berthold Technologies, Calmbacher, Germany). The transactivation ability of wild-type and mutant P53 family proteins was measured as relative light units (RLUs) and normalized first to the cultures’ absorbance (600 nm). Then the fold changes were calculated using as reference the normalized RLUs measured from cultures of each yeast reporter strains transformed with the appropriate empty vector(s) (pRS314 or pRS314 + pRS315 depending on the experiments). The relative activity from the same fold change data was calculated by comparing the activity of a protein/allele of interest with the activity of a chosen reference. Data derive from two technical replicates with at least two biological replicates except for yLFM-KSHV strain measurements (i.e., three biological replicates). 

### 2.4. Statistical Analysis

Statistical analysis was performed by using two-way ANOVA and Tukey’s multiple comparisons test or one-way ANOVA and Dunnett’s multiple comparisons test (Graphpad Prism 9) (* *p* < 0.05; ** *p* < 0.01; *** *p* < 0.001; **** *p* < 0.0001; ns: not significant). 

## 3. Results

### 3.1. The Presence of a G4 Prone Sequence Adjacent to a P53 RE Alters the Relative Activity of Wild-Type P53 Family Proteins

**Figure 1:**
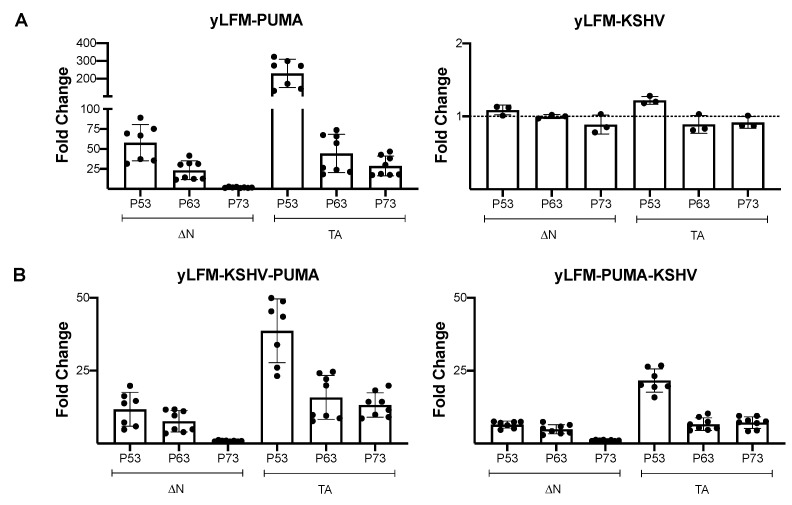
Fold change transactivation by wild-type P53 family proteins. Yeast cells expressing different P53 family isoforms by an inducible GAL1,10 promoter were grown for 8 h in Galactose 1% to evaluate their transactivation ability. (**A**) Evaluation of wild-type P53, P63, and P73 (ΔN variants: ΔN40P53α, ΔNP63α, and ΔNP73α; TA variants: full-length P53, TAP63α, and TAP73α) transcriptional activity as fold change over the empty vector in presence of the P53 response element (RE) from the *PUMA* target gene (yLFM-PUMA) or a G4 prone sequence (yLFM-KSHV). (**B**) Evaluation of transcriptional activity as in panel A in the presence of the G4 prone sequence upstream (yLFM-KSHV-PUMA) or downstream (yLFM-PUMA-KSHV) of the P53 RE from the *PUMA* target gene.

To elucidate the role of a G4 prone sequence on the transcriptional activity of P53 family members, we focused our analysis on α isoforms including ΔN40P53, full-length P53, ΔNP63, TAP63, ΔNP73, and TAP73 (ΔN40P53, ΔNP63, and ΔNP73 were indicated as ΔN variants; full-length P53, TAP63, and TAP73 were indicated as TA variants) by using a yeast-based functional assay. Consistent with previous observations regarding P53, wild-type P63 and P73 proteins (ΔN and TA variants) were unable to activate transcription (measured as fold change over empty vector) from a G4 prone sequence (yLFM-KSHV) (Figure 1A, right panel) compared to the control yLFM-PUMA (Figure 1A, left panel). Similarly, the presence of the same G4 prone sequence upstream (yLFM-KSHV-PUMA) or downstream (yLFM-PUMA-KSHV) of the PUMA P53 RE led to a significant decrease in transactivation (Figure 1B), especially in the strain where the G4 prone sequence is downstream of the RE; in this strain, the full-length P53, TAP63α, and TAP73α showed an eleven-, seven- and four-fold decrease in activity, respectively (Figure 1B, right panel).

**Figure 2:**
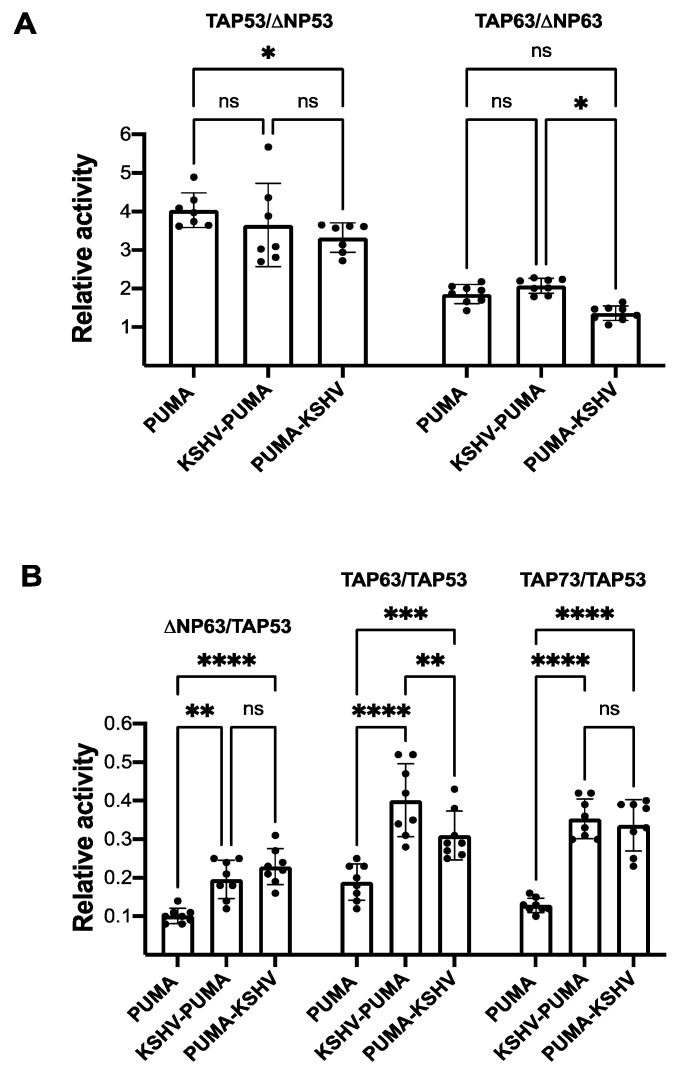
Relative activity of wild-type P53 family α isoforms. (**A**) Comparison of P53 (full-length P53) or P63 (TAP63α) TA variant activity with respect to the corresponding ΔN variant (ΔN40P53α and ΔNP63α, respectively) in yLFM-PUMA, yLFM-KSHV-PUMA, and yLFM-PUMA-KSHV strains. (**B**) Comparison of ΔNP63α, TAP63α, or TAP73α variant activity with respect to TAP53 (full-length P53) variant in yLFM-PUMA, yLFM-KSHV-PUMA, and yLFM-PUMA-KSHV strains. ns: not significant, * *p* < 0.05; ** *p* < 0.01; *** *p* < 0.001; **** *p* < 0.0001.

The results also showed that, independently from the presence of the G4 prone sequence, the TA variant from P53, P63, or P73 proteins is always significantly more active than the corresponding ΔN variant (Figure 1A,B). However, the positioning of the G4 prone sequence downstream of the P53 RE (yLFM-PUMA-KSHV) leads to a slight but significant decrease in the relative activity of TA over ΔN variants for both P53 (full-length P53/ΔN40P53α) and P63 (TAP63α/ΔNP63α) (Figure 2A). Further, by comparing across P53 family proteins, the presence of the same G4 prone sequence either upstream or downstream of the RE (yLFM-KSHV-PUMA or yLFM-PUMA-KSHV, compared to yLFM-PUMA) significantly changes all relative activities, reducing the differences in transcriptional activity between different proteins of the P53 family, as highlighted by the significant increase in ΔNP63α/full-length P53, TAP63α/full-length P53, and TAP73α/full-length P53 relative activity (Figure 2B). 

### 3.2. The Presence of a G4 Prone Sequence Adjacent to a P53 RE Determines a Variation in the Relative Functionality of Mutant P53 and P63 Proteins

Since somatic and germ-line mutations are common to the *TP53* gene in human cancers, while germ-line mutations at the *TP63* locus, mainly affecting at protein level the ΔNP63α isoform, are involved in the development of P63-associated genetic disorders, we decided to extend the functional studies to mutant P53 family proteins. To this aim, the P53 R175H and R282W proteins were selected (as full-length variants), being hot spot mutations both in sporadic cancers and Li-Fraumeni syndrome; the two mutants are also representative of a loss of function (i.e., R175H) and a partial function (i.e., R282W) mutant, respectively [42,44]. With regard to *TP63* mutations, we selected the two ΔNP63α G134V and ΔNP63α R204W variants that we identified in patients affected by P63-associated disorders and that are representative of mutant proteins with greater or lesser functionality, respectively [41]. We previously confirmed that in our yeast model the observed differences in functional features of the selected P53 or P63 mutant proteins are not due to different levels of protein expression [39,42].

**Figure 3:**
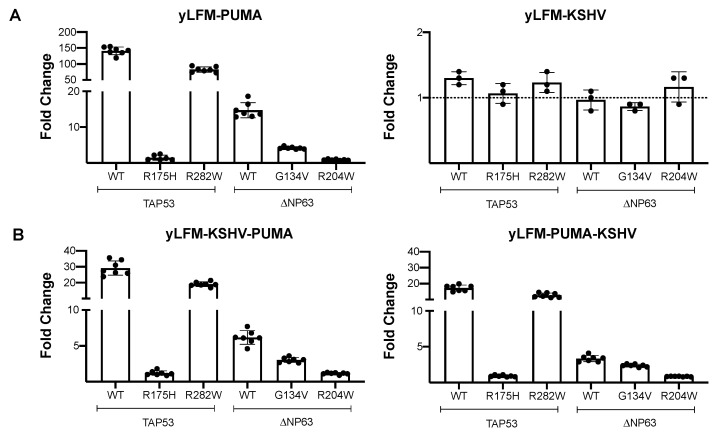
Fold change transactivation by mutant P53 family proteins. Yeast cells expressing wild-type and mutant TAP53 or ΔNP63 proteins by an inducible GAL1,10 promoter were grown for 8 h in Galactose 1% to evaluate the transactivation ability. (**A**) Evaluation of wild-type (TAP53: full-length P53; ΔNP63: ΔNP63α) or mutant (TAP53: R175H and R282W; ΔNP63: G134V and R204W) P53 and P63 proteins transcriptional activity as fold change over the empty vector in presence of the P53 RE from the *PUMA* target gene (yLFM-PUMA) or a G4 prone sequence (yLFM-KSHV). (**B**) Evaluation of transcriptional activity as in panel (**A**) in presence of the G4 prone sequence upstream (yLFM-KSHV-PUMA strain) or downstream (yLFM-PUMA-KSHV) of the P53 RE from the *PUMA* target gene. WT, wild-type.

The P53 R175H and ΔNP63α R204W proteins are characterized by consistent loss of activity in all yeast strains independently from the presence of a G4 prone sequence (Figure 3A,B). The presence of the G4 prone sequence also causes an absolute reduction in the transactivation ability of the partial function mutant TAP53 R282W and ΔNP63α G134V proteins. However, the presence of the G4 prone sequence downstream of the P53 RE significantly lowers the difference in activity with respect to the wild-type protein, especially for ΔNP63α G134V mutant (i.e., 71% of wild-type functionality in yLFM-PUMA-KSHV with respect to 28% in yLFM-PUMA) (Supplementary Figure S1).

As previously described, germ-line heterozygous mutations in the *TP53* or *TP63* gene are the molecular basis of the specific P53- and P63-related genetic disorders; then, it is conceivable that in the cells of affected patients, being P53 and P63 tetrameric TFs, wild-type, mixed, and mutant tetramers are formed. Based on this consideration, we simulated the heterozygous status in our yeast model by co-expressing wild-type and mutant P53 or P63 variants, and we evaluated the influence of the G4 prone sequence in this setting. 

**Figure 4:**
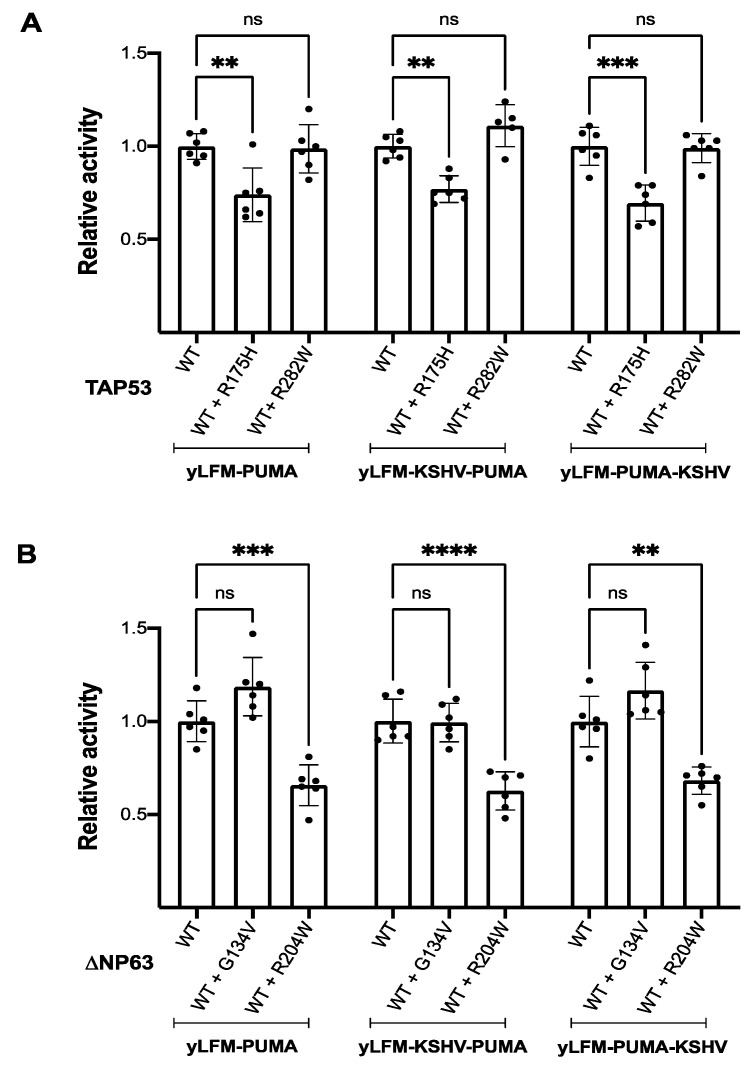
Relative activity of co-expressed wild-type and mutant TAP53 or ΔNP63 proteins compared to the corresponding single protein expression. (**A**) Comparison of wild-type and mutant TAP53 co-expression activity (i.e., constitutive pADH1-wild-type TAP53 + inducible pGAL1,10 TAP53 R175H or R282W) with that of wild-type TAP53 expressed alone (i.e., constitutive pADH1-wild-type TAP53) in presence of the P53 RE from the *PUMA* target gene (yLFM-PUMA) or the same p53 RE along with a G4 prone sequence upstream (yLFM-KSHV-PUMA) or downstream (yLFM-PUMA-KSHV). (**B**) Comparison of wild-type and mutant ΔNP63 co-expression activity (i.e., constitutive pADH1-wild-type ΔNP63 + inducible pGAL1,10 ΔNP63 G134V or R204W) with that of wild-type ΔNP63 expressed alone (i.e., constitutive pADH1 wild-type ΔNP63) in presence of the P53 RE from the *PUMA* target gene (yLFM-PUMA) or the same P53 RE along with a G4 prone sequence upstream (yLFM-KSHV-PUMA) or downstream (yLFM-PUMA-KSHV). WT, wild-type. ns: not significant, ** *p* < 0.01; *** *p* < 0.001; **** *p* < 0.0001.

Although the presence of a G4 prone sequence causes the expected reduction in transactivation ability (Supplementary Figure S2; Supplementary Figure S3), it does not alter the potential for mutant P53 or P63 proteins to inhibit the corresponding wild-type-mediated transactivation, a feature known as dominant-negative or interfering ability [45]; in fact, the P53 R175H (full-length P53) and P63 R204W (ΔNP63α) mutants that we previously described as dominant negative [41,42,43,46] retain a comparable potential to inhibit wild-type activity also in the presence of the G4 prone sequence adjacent (upstream or downstream) to the P53 RE (Figure 4A,B).

## 4. Discussion

P53 family comprises a group of potent tetrameric TFs (i.e., P53, P63, and P73) sharing a conserved DNA binding domain responsible for the binding to sequence-specific DNA REs located near promoters of the target genes. Nevertheless, the transcriptional networks regulated by these proteins are largely distinct [47]; this feature is associated with the plasticity provided by the promoters (P1 and P2) and the splicing regulation of P53 family genes (N- and C-terminal isoforms), by the modulation of cofactors and companion TFs, and by the impact of quaternary structure of P53 family proteins at promoter target sites [4,6,48].

Many studies have highlighted that RE recognition by P53 is regulated by the direct contacts with target DNA sequence; however, the so-called indirect readout provided by nucleotides within the RE that are not directly contacted by the P53 protein is also important [49,50]. This indirect readout, also referred to as “shape readout” since it appears to be related to structural properties at the P53 DNA target sites, has been recently extended to sequences surrounding the P53 RE [51]. 

Previously, we adapted a well-established yeast-based functional assay to evaluate the influence of a G4 DNA prone sequence in proximity of a P53 RE on the transactivation potential of full-length and N-terminal truncated P53 α isoforms [34]. Structurally, G4s are four-stranded nucleic acids structures held together by non-canonical Hoogsteen G-G base pairs [52,53]. G4 motifs are evolutionarily conserved from bacteria to human, confirming the importance of their formation in vivo [54]. In yeast and human, G4s are enriched in telomeric and ribosomal DNA, at transcriptional regulatory units, and at mitotic and meiotic double-strand break sites [53,55,56]. G4s can also form in RNA, where they can affect its stability or translation [57,58,59,60].

Indeed, contemporary research has demonstrated the importance of G4s in various cellular processes, including interactions with TFs [60,61,62,63]. Moreover, the targeting of the unique structure of G4s is proposed as a good tool for various diseases therapies including cancer [64,65,66,67,68]. 

Interestingly, G4s are often located in promoter sequences as shown for many human genes [69,70,71], including P53 targets; in fact, the presence of G4 prone sequences has been found around P53 RE in the promoter of the P53-induced apoptotic PUMA protein [34]. Therefore, the presence and the formation of a G4 structure could be an important transcriptional regulatory element, as previously observed in various organisms including humans [72,73,74].

In the present paper we extended for the first time the evaluation of the role of G4s in the transcription regulation to all P53 family proteins (wild-type and mutant α isoforms) by using the yeast *Saccharomyces cerevisiae* as model system; the impact of a sequence element prone to adopt a G4 secondary structure located upstream or downstream of the moderately active P53 RE from apoptotic *PUMA* target was evaluated. Moreover, the presence of G4 prone sequence is one of the possible reasons for binding of P53 to sites without a P53 RE as detected by ChIP studies [75]. However, we observed no significant activation of transcription by all analyzed proteins in presence of the G4 prone sequence alone, in agreement with the previous analysis regarding P53 protein [34]. On the contrary, we observed that the presence of the G4 prone sequence upstream, and more significantly downstream, of the RE causes inhibition of both ΔN and TA variants P53 family protein-mediated transactivation. Interestingly, despite lacking a full-length transactivation domain, ΔN isoforms are not transcriptionally inactive and have been shown to regulate the expression of their own set of genes [76,77,78,79,80,81,82,83,84,85]. The difference in relative transactivation between TA and ΔN isoforms was, however, significantly reduced by the presence of the G4 prone sequence downstream of RE, which suggests that inhibition of transcription activity by G4 in this sequence context could be variant-dependent (i.e., stronger on TA than on ΔN).

Then, we decided to extend the study to P53 family protein mutants. To this aim, P53 and P63 mutations associated with specific genetic syndromes and functionally heterogeneous in terms of transactivation ability were selected; moreover, the chosen P53 mutations are also hot spot codons in human cancers. The presence of the G4 prone sequence (especially downstream of the P53 RE) tends to lower the difference between wild-type and mutant protein transactivation activity. Conversely, the simulation of heterozygous condition of P53- and P63-associated disorders by co-expressing wild-type and mutant P53 or P63 variants failed to reveal an impact of the G4 prone sequence on the dominant negative properties of the P53 R175H and P63 R204W mutations. However, it is to be taken into consideration that, P53 family proteins acting as tetramers, there is a variation in the modality by which tetramers are constituted and in the possible stoichiometry of hetero-tetramers comprising the combination of wild-type and missense mutations; thus, the adaptability of our yeast assay will allow further insights in this direction. 

## 5. Conclusions

Overall, our data suggest that G4 prone sequences proximal to a P53 RE lead to an overall reduction of P53 family proteins-dependent transactivation. Still, at the same time, their presence can modulate the interplay between isoforms within the same protein and between different members of P53 family. Moreover, some functional features of mutant P53 family proteins can also be affected by the placement of a G4 prone sequence adjacent to a P53 RE. Hence, we propose that the sequence context surrounding a P53 RE can contribute to tuning P53 family proteins functions and should be considered as an important variable to fully characterize the P53 family cistrome. Furthermore, given that the net transcriptional effect of the P53 family proteins can be dependent on the ratio TA/ΔN variants of P53, P63, and P73 isoforms and on wild-type and mutant P53/P63/P73 interactions and binding to the promoters of target genes, our finding also paves the way for future studies involving the use of available small molecules that can modulate the conformations of G4s structure-forming DNA sequences [86].

Lastly, the yeast *Saccharomyces cerevisiae* has reconfirmed itself as a robust model system that can be turned into a sort of in vivo test-tube for the functional analysis of human TFs. Although the placement of a G4 prone sequence adjacent to a P53 RE within a minimal promoter can impact the site’s global transactivation potential, our approach in yeast mainly focuses on the relative effect towards specific P53 family proteins or on their functional interactions. The otherwise isogenic nature of the reporter yeast strains and the regulated systems for ectopic protein expression we used give us confidence that the observed relative differences are not dependent on other variables.

## Data Availability

Not applicable.

## Supplementary Materials

The following are available online at https://www.mdpi.com/2073-4425/12/2/277/s1, Figure S1: Relative activity of mutant P53 and P63 proteins, Figure S2: Fold change transactivation by co-expressed wild-type and mutant P53 proteins, Figure S3: Fold change transactivation by co-expressed wild-type and mutant P63 proteins.
