# Supplementary material for: Evaluating the Influence of a G-Quadruplex Prone Sequence on the Transactivation Potential by Wild-Type and/or Mutant P53 Family Proteins through a Yeast-Based Functional Assay
Source: Genes (Basel). 2021 Feb 15;12(2):277. doi: 10.3390/genes12020277 (PMC7919268; doi:10.3390/genes12020277)
Supplement: Supplementary file 1 [file genes-12-00277-s001.pdf]

Supplementary Materials

Evaluating the influence of a G-quadruplex prone sequence on the transactivation potential by wild-type and/or mutant P53 family proteins through a yeast-based functional assay.

Paola Monti[1], Vaclav Brazda[2,3], Natália Bohálová[2,4], Otília Porubiaková[2,3], Paola Menichini[1], Andrea Speciale[1], Renata Bocciardi[5,6] Alberto Inga[7*] and Gilberto Fronza[1*]

[1] Mutagenesis and Cancer Prevention Unit, IRCCS Ospedale Policlinico San Martino, Genoa 16132, Italy; paola.monti@hsanmartino.it (P.M.); paola.menichini@hsanmartino.it (P.M.); andrea.speciale@hsanmartino.it (A.S.)

[2] Institute of Biophysics of the Czech Academy of Sciences, Královopolská 135, 61265 Brno, Czech Republic; vaclav@ibp.cz (V.B.); natalia.bohalova@ibp.cz (N.B.); o.porubiakova@gmail.com (O.P.)

[3] Department of Food Chemistry and Biotechnology, Faculty of Chemistry, Brno University of Technology, Purkyňova 118, 61200 Brno, Czech Republic

[4] Department of Experimental Biology, Faculty of Science, Masaryk University, Kamenice 5, 62500 Brno, Czech Republic

[5] Department of Neurosciences, Rehabilitation, Ophthalmology, Genetics, Maternal and Child Health (DiNOGMI), University of Genoa, Largo P. Daneo, 3, 16132 Genoa, Italy; bocciardi@unige.it (R.B.)

[6] Medical Genetics Unit, IRCCS Istituto Giannina Gaslini, via G. Gaslini, 5, 16147 Genoa, Italy

[7] Laboratory of Transcriptional Networks, Department of Cellular, Computational and Integrative Biology, CIBIO, University of Trento, via Sommarive 9, 38123 Trento, Italy

* Correspondence: alberto.inga@unitn.it (A.I.); gilberto.fronza@hsanmartino.it (G.F.); Tel.: +39-0461-283714 (A.I.); +39-010-5558225 (G.F.)

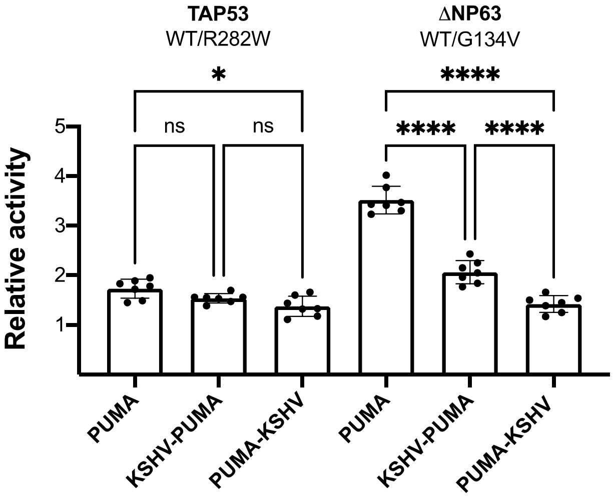

Supplementary Figure S1. Relative activity of mutant P53 and P63 proteins. Comparison of wild-type TAP53 (full-length P53) or ΔNP63 (ΔNP63α) variant activity with respect to the corresponding mutant protein (TAP53: R175H and R282W; ΔNP63α: G134V and R204W) in yLFM-PUMA, yLFM-KSHV-PUMA, and yLFM-PUMA-KSHV strains. WT, wild-type. ns: not significant; ,* p < 0.05; ** p < 0.01; *** p < 0.001; **** p < 0.0001

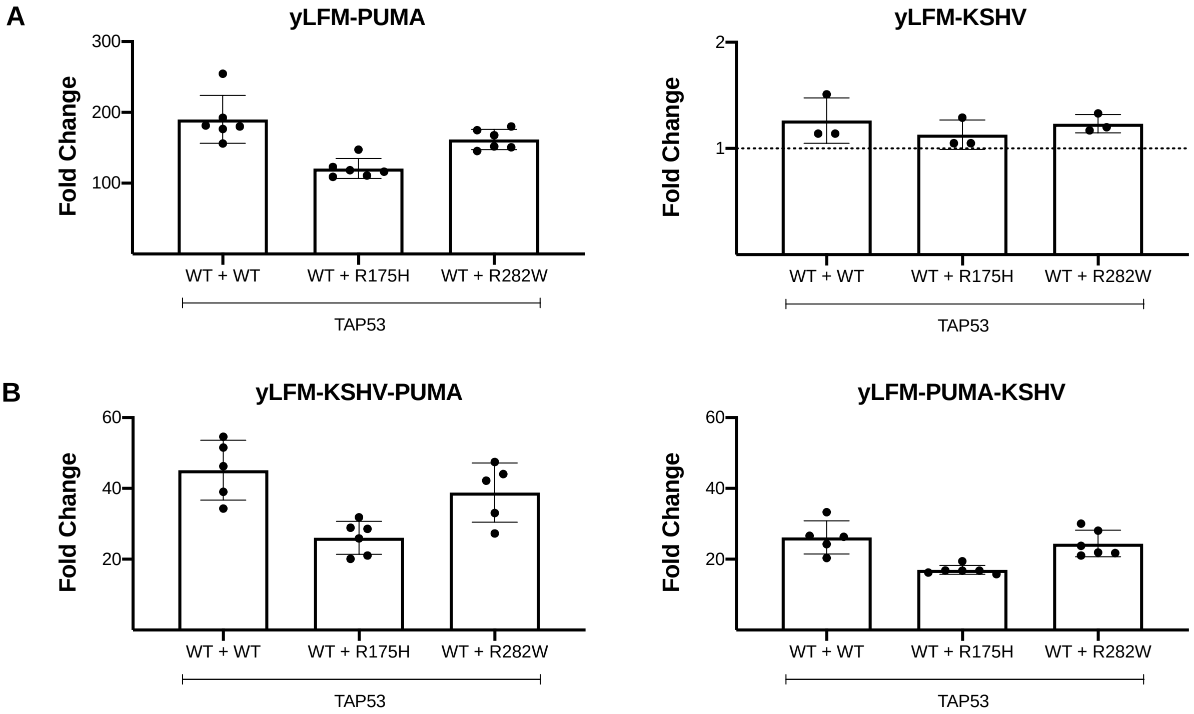

Supplementary Figure S2. Fold change transactivation by co-expressed wild-type and mutant P53 proteins. Yeast cells co-expressing wild-type or wild-type and mutant TAP53 proteins were grown for 8 hours in Galactose 1% to evaluate the transactivation ability. **(A)** Evaluation of transcriptional activity from wild-type (i.e., constitutive pADH1-wild-type TAP53 + inducible pGal1,10-wild-type TAP53) or wild-type and mutant P53 (i.e., constitutive pADH1-wild-type TAP53 + inducible pGal1,10 TAP53 R175H or R282W) co-expression as fold change over empty vectors in presence of the p53 RE from the *PUMA* target gene (yLFM-PUMA) or a G4 prone sequence (yLFM-KSHV). **(B)** Evaluation of transcriptional activity as in panel A in presence of a G4 prone sequence upstream (yLFM-KSHV-PUMA) or downstream (yLFM-PUMA-KSHV) of the p53 RE from the *PUMA* target gene. WT, wild-type.

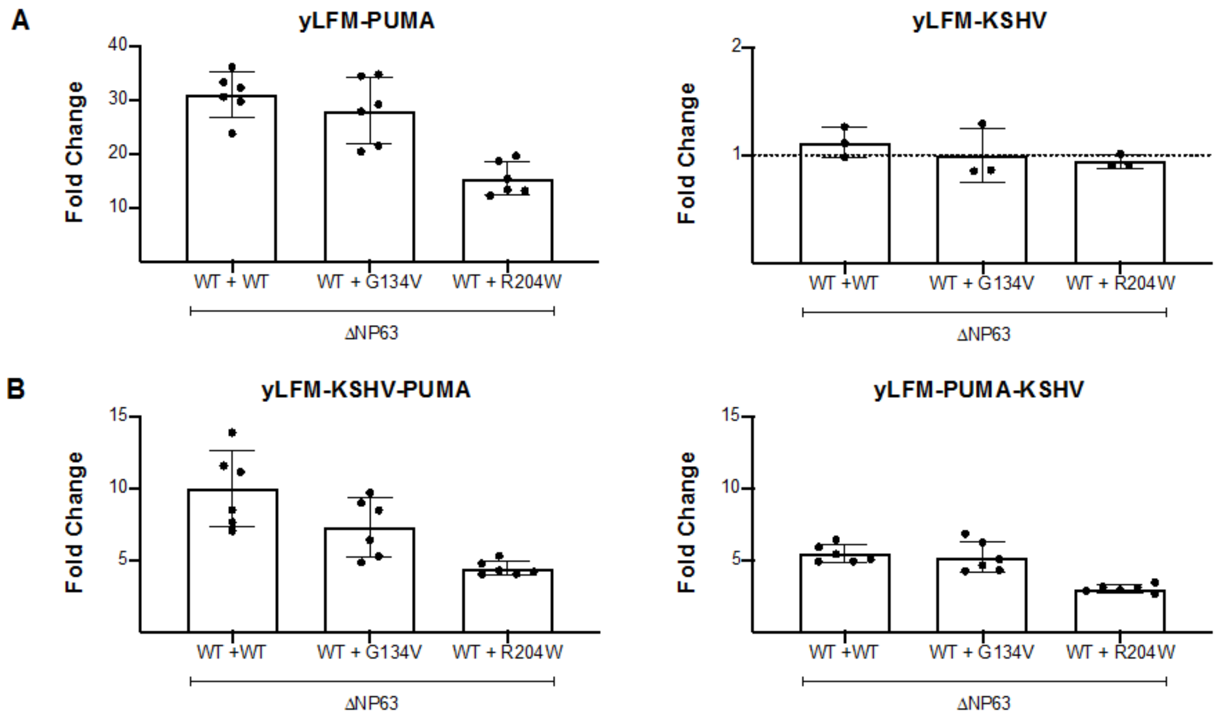

Supplementary Figure S3. Fold change transactivation by co-expressed wild-type and mutant P63 proteins. Yeast cells co-expressing wild-type or wild-type and mutant ΔNP63 proteins were grown for 8 hours in Galactose 1% to evaluate the transactivation ability. **(A)** Evaluation of transcriptional activity from wild-type (i.e., constitutive pADH1-wild-type ΔNP63 + inducible pGal1,10-wild-type ΔNP63) or wild-type and mutant P63 (i.e., constitutive pADH1-wild-type ΔNP63 + inducible pGal1,10 ΔNP63 G134V or R204W) co-expression as fold change over empty vectors in presence of the p53 RE from the *PUMA* target gene (yLFM-PUMA) or a G4 prone sequence (yLFM-KSHV). **(B)** Evaluation of transcriptional activity as in panel A in presence of a G4 prone sequence upstream (yLFM-KSHV-PUMA) or downstream (yLFM-PUMA-KSHV) of the p53 RE from the *PUMA* target gene. WT, wild-type
